# Molecular and Functional Properties of Protein Fractions and Isolate from Cashew Nut (*Anacardium occidentale* L.)

**DOI:** 10.3390/molecules23020393

**Published:** 2018-02-12

**Authors:** Cheng-mei Liu, Qian Peng, Jun-zhen Zhong, Wei Liu, Ye-jun Zhong, Fang Wang

**Affiliations:** State Key Laboratory of Food Science and Technology, Nanchang University, Nanchang, No. 235 Nanjing East Road, Nanchang 330047, China; liuchengmei@aliyun.com (C.-m.L.); ncupengqian@163.com (Q.P.); zhongjunzhen@163.com (J.-z.Z.); liuwei@ncu.edu.cn (W.L.); ncuskwfang@163.com (F.W.)

**Keywords:** cashew nut, protein fraction, functional properties, physicochemical characterization

## Abstract

Some molecular and functional properties of albumin (83.6% protein), globulin (95.5% protein), glutelin (81.3% protein) as well as protein isolate (80.7% protein) from cashew nut were investigated. These proteins were subjected to molecular (circular dichroism, gel electrophoresis, scanning electron microscopy) and functional (solubility, emulsification, foaming, water/oil holding capacity) tests. Cashew nut proteins represent an abundant nutrient with well-balanced amino acid composition and could meet the requirements recommended by FAO/WHO. SDS-PAGE pattern indicated cashew nut proteins were mainly composed of a polypeptide with molecular weight (MW) of 53 kDa, which presented two bands with MW of 32 and 21 kDa under reducing conditions. The far-UV CD spectra indicated that cashew proteins were rich in β-sheets. The surface hydrophobicity of the protein isolate was higher than that of the protein fractions. In pH 7.0, the solubility of protein fractions was above 70%, which was higher than protein isolate at any pH. Glutelin had the highest water/oil holding capacity and foaming properties. Protein isolate displayed better emulsifying properties than protein fractions. In summary, cashew nut kernel proteins have potential as valuable nutrition sources and could be used effectively in the food industry.

## 1. Introduction

The cashew tree (*Anacardium occidentale* L.) is native to the Brazil and now widely cultivated throughout the world. Cashew nut is one of the four famous nuts in the world and plays an important role for its products with high nutritional value and unique flavor. Cashew nut in a healthy diet can not only lower the risk of cardiovascular disease, especially stroke, but also reduce the risk of metabolic syndrome [1]. Cashew nut kernel has a composition of 40–57% lipid, 23–25% carbohydrates and 20–25% high quality protein content, of the lipid, 61% is monounsaturated fatty acids and 17% is polyunsaturated fatty acids [2,3,4]. Defatted cashew flour has been indicated as a good protein source with potential applications due to its higher protein content [5]. Cashew nut protein is new but nutritional, previous study showed cashew nut protein had well-balanced in essential amino acids [6]. The 13S globulin of cashew nut was composed of two major types polypeptides with molecular weights in the range of 18,000–24,000 and 30,000–37,000 Da [7]. To our knowledge, the composition, varieties and second structure of cashew nut protein have not been reported.

Previous studies have focused on cashew protein isolate and concentrate. According to Ogunwolu et al. [8], the functional properties of cashew protein concentrate and isolate were significantly different (*p* < 0.05), and the functional properties of cashew nut proteins were comparable to soybean, lupin and peanut protein, which are already being used in many food products as functional ingredients. Their study also indicated that protein isolate and concentrate had superior water/oil absorption capacity and foaming capacity than defatted flour. Neto et al. [9] reported the functional properties of protein isolate from raw and heat processed cashew nut were different, and what’s more, the functional properties showed excellent correlation with solubility [10]. Previous findings also showed that globulin was the major protein fraction of cashew nut. However, there is scant information on the different cashew nut protein fractions corresponding to these functional and molecular properties.

Recently, plant proteins are attracting more and more interest due to their digestible and nutritional character. With the increasing demand for plant proteins, great efforts have been aimed at taking full advantage of new plant proteins as food ingredients, such as soapnut seed proteins [11], pumpkin seed proteins [12], mung bean proteins [13], bitter melon seed proteins [14], etc. Cashew nut protein, with abundant nutritional and great economic value, requires more detailed functional and molecular data. Therefore, this study attempted to determine the molecular and functional properties of albumin, globulin, glutelin as well as protein isolate from cashew nut, which may provide opportunities to effectively utilize these proteins as functional food ingredients.

## 2. Results

### 2.1. Chemical Compositions

The chemical compositions of cashew nut protein isolate (CNPI), defatted cashew nut flour (DCNF), albumin, globulin, glutelin and prolamin are listed in Table 1. The protein content, moisture, crude fat, ash and soluble sugar in DCNF were 31.9%, 9.24%, 0.5%, 4.46% and 19.15%, respectively. This result indicated that DCNF was rich in protein. The crude protein contents of albumin, globulin, glutelin and CNPI were 83.6%, 95.5% and 81.3%, higher than CNPI (80.7%). What’s more, the soluble sugar content of all samples was lower than 3.23%, the crude fat of globulin and glutelin even lower than 1%, which indicated the Osborne fractionation method was practicable to preliminarily purify cashew nut protein. A similar result was observed in the study of Tang and Wang [15] where buckwheat globulin (96.7%) had higher protein content than albumin (57.0%). The differences in protein content suggested the water-soluble protein had other minor components such as soluble sugars and polyphenols. These results revealed that it was practical to sequentially extract proteins from cashew nut using water, salt and alkali solution, which should promote the economical industrial processing of cashew nut proteins [16]. As shown in Table 1, the gross yields of albumin, globulin and glutelin were 7.69%, 17.30% and 7.80% (*w/w* of flour), total output was higher than CNPI (19.67%). The Osborne solubility-based protein extraction method indicated that water-soluble (albumin), NaCl-soluble (globulin) and NaOH-soluble (glutelin) were the main fractions in cashew nut protein, accounting for 22.87%, 51.44% and 23.19% (*w/w*), respectively, while only 2.5% of ethanol-soluble fraction (prolamin) was obtained, hence, in the following research we will investigate in the properties of albumin, globulin and glutelin from cashew nut kernel.

The amino acid composition of cashew nut proteins is presented in Table 2, where the recommended FAO/WHO (2007) patterns for children and adults were taken as reference. As it shows, the amino acid composition of albumin, globulin, glutelin and CNPI exhibited distinct differences. The four samples are all rich in valine, isoleucine and cysteine, while the glutamic and arginine content of albumin and globulin were significant higher than in glutelin, accounting for over 37.87%. According to the FAO/WHO/UNU recommended essential amino acid patterns, the valine, isoleucine and lysine levels of the four protein samples could satisfy the children’s requirement, that means cashew nut proteins could be used as a replenisher in food for babies or young children. Furthermore, the essential amino acid content of cashew nut protein fractions was higher than CNPI, especially in glutelin, where the ratio of essential to total amino acids was as high as 41.11%. According to a previous study, sulphur amino acids are the limiting amino acid in legume seed proteins [17], but our study showed that albumin and globulin could be used as an excellent source of supplements. Comparing with albumin and globulin, glutelin has higher aromatic and hydrophobic amino acid content, which could enhance protein-protein interactions and result in higher oil holding capacity. Albumin had a lower aromatic and hydrophobic amino acid content that may explain its higher solubility. The ratio of arginine and lysine was higher in globulin than albumin and glutelin, indicating a better nutritional property of globulin because a high arginine to lysine ratio may reduce the risk of cardiovascular disease [18].

### 2.2. Molecular Characterization

#### 2.2.1. Electrophoresis

**Figure 1:**
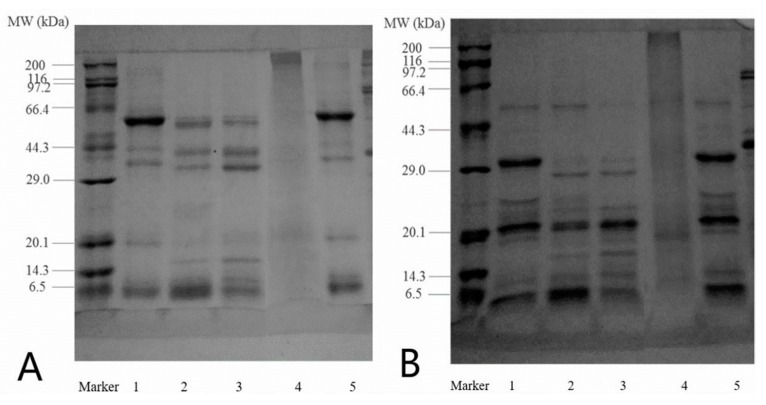
Non-reducing (**A**) and reducing (**B**) SDS-PAGE profiles of cashew nut proteins. (Lane 1, DCNF; Lane 2, albumin; Lane 3, globulin; Lane 4, glutelin; Lane 5, CNPI). The molecular weights of markers are 6.5, 14.3, 20.1, 29.0, 44.3, 66.4, 97.2, 116 and 200 kDa, respectively.

The SDS-PAGE profiles of raw cashew nut protein, CNPI, albumin, globulin and glutelin under non-reducing (A) and reducing conditions (B) are presented in Figure 1. Under non-reducing conditions, the CNPI and DCNF showed a large similarity with the 53 kDa being in the highest proportion. There were five main polypeptides with the estimated molecular weight (MW) of 6.5, 19, 34, 44, 53 kDa, the MW distribution was similar to that reported in a previous study by Sathe et al. [19]. For the albumin and globulin, there were additionally two polypeptides with MW of 16 and 10 kDa. The highest proportion of albumin was around 7 kDa while in globulin was 34 kDa. Glutelin exhibited three weak bands with MW about 15, 20 and 43 kDa, while the stain was intense between the separation and stacking gel. This indicated that glutelin may have bigger MW and lower solubility in gel buffer, and most of glutelin cannot enter the gel [20]. After adding β-mercaptoethanol, the subunit composition of all protein samples presented marked changes. For all samples, the 53 kDa polypeptide band narrowed and presented two bands with MW of 32 and 21 kDa. The polypeptide of 53 kDa was barely visible in globulin but was quite clear in albumin, which was consistent with previous work by Sathe [21]. Meanwhile the polypeptide of 44 and 34 kDa disappeared and the band of 27 kDa was darker in albumin and globulin, evidencing that the polypeptides contained inter-molecular disulfide bonds. The disappearance of high MW protein with the presence of β-ME had been reported in hemp seed proteins [22]. What’s more, there were several small polypeptides with MW less than 14 kDa. Compared with albumin and globulin, glutelin had a larger molecular weight.

#### 2.2.2. Circular Dichroism Spectroscopy

In the study, far-UV circular dichroism (CD) spectroscopy was used to determine the secondary structure composition of cashew nut proteins. The quantitative estimation of the relative amounts is shown in Table 3. The secondary structure of albumin was composed of 9.5% α-helix, 36.4% β-sheet, 18.6% turns and 35.5% random coils. Globulin was composed of 8.6% α-helix, 41.1% β-sheet, 22.5% turns and 29.5% random coils. The α-helix proportion of glutelin was 11.7%, the other was similar to albumin. CNPI had a similar content of turns and random coils as globulin, but more α-helix and less β-sheets. The secondary structures of cashew nut proteins were mainly β-sheets and random coils. Since the α-helix, β-sheet and turns were found to account for about 70%, the globulin and CNPI could be considered as proteins with highly ordered and stable conformations [23,24]. The higher proportion of turns in CNPI suggested that this protein unfolded and dissociated during the acid precipitation process [25]. Furthermore, Marcone et al. [26] showed that plant globulins were rich in β-sheet, such as amaranth globulin (46.3%), soybean globulin (42.2%) and wheat 11S globulin (53.3%). Yin et al. [11] also reported that protein fractions from soapnut contained predominantly β-sheet structures. Compared with globulin, higher amounts of α-helix and random coils were observed in albumin, indicating a more flexible conformation.

#### 2.2.3. Surface Hydrophobicity (H_0_)

**Figure 2:**
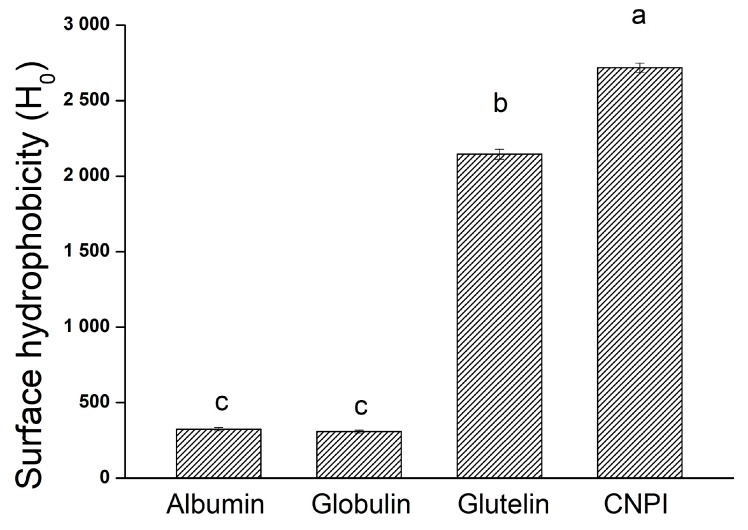
Surface hydrophobicity of albumin, globulin, glutelin and CNPI from cashew nut. Columns with different letters (**a**–**c**) are significantly different (*p* < 0.05), as analyzed by the SPSS software.

The surface hydrophobicity value reflects the extent of exposure of the hydrophobic clusters on the protein molecule surface and is related to its interfacial activity. Figure 2 shows that the H_0_ values of CNPI and glutelin were 2717.81 and 2145.18, respectively, which were significant higher (*p* < 0.05) than albumin (324.40) and globulin (308.76). The phenomenon was consistent with the result obtained in Section 2.3 whereby albumin and globulin had better solubility in neutral pH. A lower H_0_ value indicated there was less hydrophobic groups on the protein surface, therefore resulting in higher solubility. The higher H_0_ of glutelin and CNPI may be attributable to their flaky and irregular microstructures. Proteins with high H_0_ values tend to exhibit greater levels of surfactancy, which character may result in excellent foaming and emulsifying capacity [27].

#### 2.2.4. Scanning Electron Microscopy

**Figure 3:**
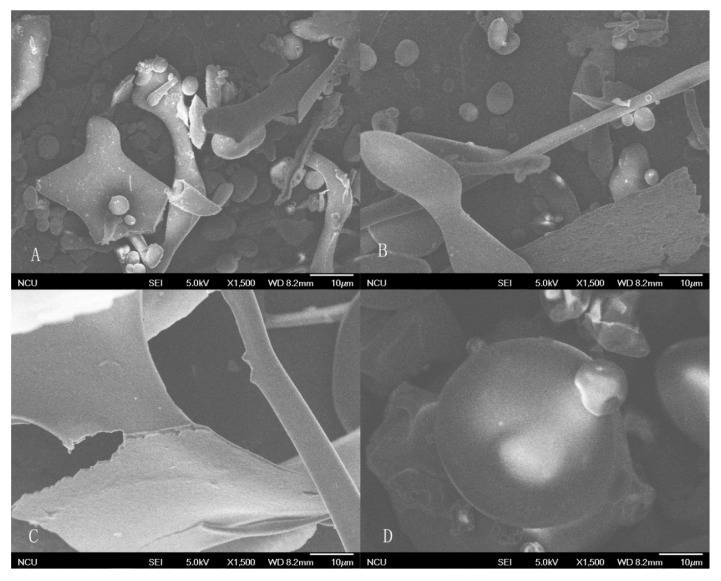
The SEM images of cashew nut albumin (**A**); globulin (**B**); glutelin (**C**) and CNPI (**D**).

The SEM images of cashew nut proteins are shown in Figure 3. Albumin and globulin displayed obvious globular structures combined with some rhabditiform and irregular lamellar parts. Glutelin showed a big flaky structure with a smooth surface and no globular structures were observed. On the other hand, CNPI show bigger globules with less irregular shape and some indentations. The flaky structure of glutelin may be due to the strong alkali environment, and the loose and lamellar structure after extraction is similar to the result of other physical and chemical modifications, such as enzymatic hydrolysis [28], high-pressure homogenization and succinylation [29], and these microstructure changes may result in the exposure of hydrophobic amino acid residues and the alteration of protein functional properties. The particle size of CNPI was bigger and with surface depressions because the acid treatment process may lead to aggregation and the formation of a more compact structure.

### 2.3. Protein Solubility (PS)

**Figure 4:**
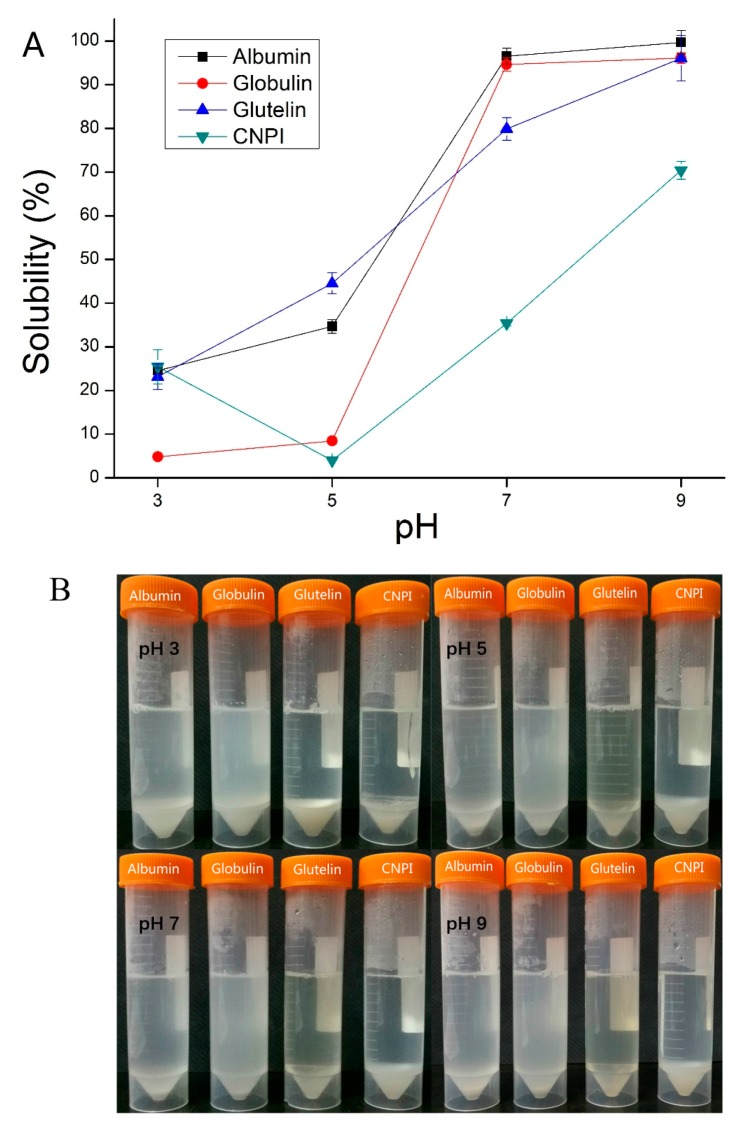
Solubility-pH profiles (**A**) and photograph of solubility (**B**) of cashew nut protein fractions compare with CNPI in different pH (3, 5, 7, 9).

Protein solubility plays a key role among the functional properties because the protein should initially form solutions before other propertiesare performed [30]. As shown in Figure 4, the solubility of cashew nut proteins was found to be extremely sensitive to pH, and the solubility of protein fractions increased with increasing pH, while the solubility-pH profile of CNPI shows a U curve shape. The minimum solubility of cashew nut proteins was observed around pH 3.0 and 5.0, which might be due to the increased protein-protein interactions near the isoelectric point, while the maximum protein solubility was observed at pH 9.0. In a neutral environment, the solubility of albumin and globulin was 96.55% and 94.65%. Similar results have been reported for the solubility of cashew nut protein isolate (95%) and concentrate (95%) [8]. The solubility of glutelin also reached to 79.88%, but the PS of CNPI in our study was only 35.36%. The PS of all samples were significantly higher (*p* < 0.05) in neutral and alkaline environments, a phenomenon that is consistent with hemp seed protein fractions [22]. The excellent protein solubility indicated cashew protein fractions had potential practical applications in the food industry, since most food is produced in neutral or weak acidic/alkaline environments. The PS of cashew nut protein fractions in a neutral environment was higher than that of other plant protein fractions, such as African yam bean protein [31], hemp seed protein, *Torreya grandis* seeds protein [16] and kidney bean protein [17]. The higher solubility of albumin could be attributed to its higher soluble sugar content (2.03%), as carbohydrate components may enhance protein-water interactions and thus increase the solubility [17]. The solubility pattern indicated that cashew nut protein fractions could be adequate for most food formulations.

### 2.4. Water and Oil Holding Capacity

Water holding capacity (WHC) is described as the ability of a protein to retain water against gravity, including hydrodynamic water, bound water, capillary water and physically entrapped water [32]. The WHC of cashew nut albumin, globulin, glutelin and CNPI is listed in Table 4. Glutelin had the highest WHC of 15.85 g/g, significantly higher (*p* < 0.05) than CNPI (1.75 g/g), albumin (1.25 g/g) and globulin (0.95 g/g). The high WHC of glutelin might be due to its flaky microstructure that enhanced its interactions with water. The WHC values of cashew nut proteins in the study were comparable to Chilean hazelnut protein (1.34 g/g) [33], albumin (0.41 g/g) and globulin (1.51 g/g) from ginkgo seed [34]. Glutelin and CNPI, with their high WHC values, are considered as a potential food ingredients in viscous food [35].

Oil holding capacity (OHC) plays an important role in food flavor retention and food formation, especially for the meat industry, and also affects the emulsifying capacity. Glutelin was found to have the highest oil holding capacity value of 27.47 g/g compared with that of globulin (9.34 g/g), albumin (5.94 g/g) and CNPI (1.05 g/g). The OHC of glutelin was higher than a previous study of cashew nut protein concentrate (3.32 g/g) and isolate (4.42 g/g) [8], indicating cashew nut protein fractions obtained by the Osborne extraction method may have more outstanding functional properties than protein isolate and concentrate. These results were also comparable to the value of 1.7 g/g for chickpea protein isolate [36], 4.75 g/g of globulin from African yam bean seed [22] and 3.0 g/g for sunflower protein concentrate [37]. Glutelin had higher OHC and WHC, indicating it might be a kind of natural amphiphilic substance, while the other protein samples contain more β-sheet and turn structures that may limit their interaction with water and lipid phases. Glutelin has a more open structure that may be another reason for its prominent water/oil holding capacity. Proteins possess excellent surfactant character and for this reason can be widely utilized in the food industry, since oil and water holding capacity is essential in food processing [38]. Ogunwolu et al. [8] reported that OHC increased with purity in protein products, which may be the reason why the OHC of globulin was higher than that of albumin.

### 2.5. Emulsifying and Foaming Properties

The emulsion activity index (EAI) represents the ability of a protein to form emulsions via absorbing at the oil water interface. The EAI can be influenced by many factors, including protein concentration, pH and the presence of non-protein compounds. The emulsifying properties of the cashew nut protein fractions in a neutral environment are shown in Table 5. CNPI had a distinctly higher EAI (20.21 m^2^/g), followed by albumin (10.46 m^2^/g), globulin (7.98 m^2^/g) and glutelin (7.03 m^2^/g), suggesting water-soluble and acid precipitation proteins could stabilize a higher interfacial area. However, the EAI of CNPI was higher than that of canola protein isolate (15.0 m^2^/g) in previous work from Karaca et al. [39], and cashew nut proteins reported by Ogunwolu et al. [8]. Thus, cashew nut protein isolate was comparable to other plant proteins in EAI and suitable for emulsified foods such as ice cream, baked sweets and sausages. The EAI of albumin was significant higher (*p* < 0.05) than that of glutelin, and similar with the results of *Akebia trifoliata* var. *australis* seed protein fractions [20]. Some studies have reported that emulsifying properties can be influenced by surface hydrophobicity and solubility mutually or solely [33]. According to Kato and Nakai [40], proteins with more hydrophobic groups could decrease interfacial tensions and increase the emulsifying capacity. Higher EAI of CNPI may be attributed to high surface hydrophobicity while in the case of albumin it may be due to its excellent solubility.

The term emulsion stability (ES) represents the ability of emulsions to resist physiochemical changes over time. In general, CNPI showed significantly higher emulsion stability (61.78 min), followed by albumin (41.84 min), globulin (28.08 min) and glutelin (21.57 min), respectively. CNPI produced by alkali extraction and acid precipitation not only excelled in emulsion formation, but also was more stable than other proteins, while albumin with higher solubility and lower surface hydrophobicity could be responsible for the higher emulsion stability value. The result was consistent with the results for hemp [22] and ginkgo seed [34] proteins in previous studies. Emulsion stability of cashew nut protein was superior to protein from flaxseed (12.51 min) [41], chickpea (10.92 min), faba bean (10.97 min) and pea (12.40 min) [42]. It’s generally known that emulsions are highly popular in the food industry and suitable emulsifiers are helpful for creating foods with desirable qualities [43]. The superior solubility and relatively better emulsification properties of albumin and CNPI might be advantageous in food production.

**Figure 5:**
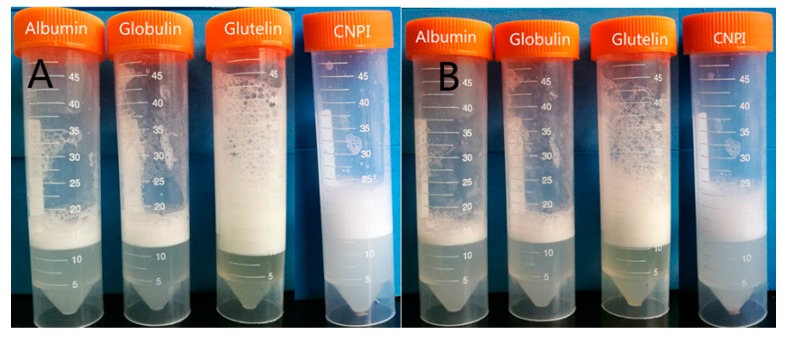
Foaming property of cashew nut albumin, globulin, glutelin and CNPI. The photograph of foaming volume after 0 min (**A**) and 30 min (**B**).

The foaming capacity (FC) and foam stability (FS) of cashew nut proteins are shown in Table 5. In the natural pH environment, the foaming capacity of glutelin and CNPI was 101.93% and 92.00%, respectively, more than that of globulin (54.05%) and albumin (20.48%). As shown in Figure 5A, glutelin has the highest foaming capacity, as its excellent amphiphilic property enhance interactions with the aqueous phase that then possesses better capacity to encapsulate air particles. Globulin with high purity can form higher protein concentration solutions, though it can easily unfold and enhance protein-protein interactions to form stronger protein interfacial membranes, then showing better foaming capacity [44]. Like foaming capacity, the foam stability of glutelin and CNPI was 79.18% and 76.70%, respectively, higher than globulin (46.00%) and albumin (19.50%). As shown in Figure 5B, the foam volume after 30 min was decreased to different degrees. This is an important property in the food industry since the effectiveness of whipping agents depends upon their ability to retain the whip as long as possible [45]. Glutelin has excellent foaming properties that may be attributed to its high content of hydrophobic amino acids, which easily to form stronger interfacial membranes between the air-water interface via greater protein-protein interactions. Overall, glutelin and DCNF can be considered as appropriate foaming ingredients in food production, such as ice creams, bakery products and drinks.

## 3. Materials and Methods

### 3.1. Materials

Shelled raw cashew nut (*Anacardium occidentale* L.) was purchased from the local HongCheng Market in Nanchang (Nanchang, Jiangxi, China) and stored at −4 °C until use. Gel electrophoresis reagent, molecular weight protein markers (14.4–116.0 kDa) and Folin-phenol reagent were purchased from Solarbio Science & Technology Co., Ltd. (Beijing, China). Sodium dodecyl sulfate (SDS), bovine serum albumin (BSA), and Coomassie Brilliant Blue R-250 were supplied by Sangon Biotech Co., Ltd. (Shanghai, China). All other chemicals and reagents used were of analytical grade or better.

### 3.2. Preparation of Defatted Cashew Nut Flour, Protein Isolate and Fractions

Raw cashew nuts were ground using a grinder and defatted with petroleum ether (boiling range: 60–90 °C) at a material/solvent ratio of 1:10 by extracting for 22 h. The dispersion was filtered through a Buchner funnel at ambient temperature and the residue used for the next extraction. The defatting procedure was repeated three times. Finally, the defatted flakes were air-dried in a fume hood until the solvent was removed completely, then ground to a powder to obtain defatted cashew nut flour (DCNF).

Cashew nut protein isolate (CNPI) was prepared according to the process described by Skevkani et al. [46]. Briefly, the defatted cashew nut flour was dispersed in deionized water (1:10, *w/v*) and the pH was adjusted to 9.0 using 1 M NaOH. After magnetic stirring for 2 h at room temperature (about 25 °C), the suspension was centrifuged at 6000× *g* for 30 min. Then the supernatants were collected, the sediments were resuspended in deionized water to repeat the extraction as described above. The two supernatants were pooled and the pH adjusted to 4.6 using 1 M HCl. The precipitated proteins were recovered by centrifugation for 30 min then resuspended in deionized water, neutralized, and subjected to dialysis (6–8 kDa cut off) against water at 4 °C for 72 h. After freeze-drying, the CNPI was stored at 4 °C until use.

The protein fractions of cashew nut were extracted using the Osborne method as follows [34]. The defatted cashew nut flour was dispersed in deionised water (1:10, *w/v*) and stirred for 2 h at room temperature. The suspension was then centrifuged at 6000× *g* for 30 min and the supernatant was collected as the albumin extract. The precipitate was extracted with 1 M NaCl (1:10, *w/v*) for 2 h and centrifuged to give the globulin fraction. The residue was then extracted with 70% ethanol to give prolamin, followed by glutelin extraction with 0.1 M NaOH. To further clarify the supernatant of insoluble residues, each extraction was performed twice. Then the protein fractions were dialyzed (6–8 kDa cut off) against water at 4 °C for 72 h, freeze-dried and stored at 4 °C.

### 3.3. Chemical Analysis

The content of crude protein, crude fat, ash, fiber and moisture of protein samples were determined by the standard 1990 AOAC methods of analysis.

#### 3.3.1. Protein Solubility (PS)

Protein solubility was determined by the method of Lowry with minor modifications [47]. Briefly, protein samples (50 mg) were dispersed in deionized water (25 mL) followed by stirring 30 min at ambient temperature (25 °C), the pH of suspensions was adjusted to the desired value (3.0–9.0) with 0.1 M HCl or NaOH. These suspensions were magnetically stirred for another 30 min then centrifuged at 7000× *g* for 15 min. The total protein content was measure by dissolving the CNPI or CNSPI in 0.1 M NaOH. PS was expressed as the percentage ratio of supernatant protein content to the total protein content.

#### 3.3.2. Oil and Water Holding Capacity

The oil and water holding capacity (O/WHC) were determined by the method of Sze-Tao and Sathe [48] with some modifications. Protein samples (0.5 g) were mixed with soybean oil or deionized water (5 mL) in a 50 mL pre-weighed centrifuge tube. The mixture was vortexed for 1 min, left standing at room temperature for 30 min then centrifuged 10 min at 2000 r/min. The supernatant was decanted, excess oil (or water) in the upper phase drained for 30 min and the sample weighed again to determine the percentage of oil or water retained per gram of sample.

#### 3.3.3. Foaming Capacity and Foam Stability

Foaming capacity (FC) and foam stability (FS) of the proteins were determined using the modified methods of Lassissi et al. [49] and Liu et al. [50]. Protein samples (50 mg) were weighed and dispersed in 0.01 M phosphate buffer (10 mL) with a pH of 7.0 (*V*_0_). Then they were homogenized with an Ultra-Turrax T 25 (IKA-Labortechnik, Staufen, Germany) at 10,000 rpm for 2 min, poured into 50 mL graduated cylinders and the foam volume recorded at 0 min (*V*_1_) and 30 min (*V*_2_). FC and FS were calculated by the following Equation:

(1) $$FC=\frac{V_{1}-V_{0}}{V_{0}}\times 100\%$$

(2) $$FS=\frac{V_{2}-V_{0}}{V_{0}}\times 100\%$$

#### 3.3.4. Emulsifying Activity Index (EAI) and Emulsion Stability (ES)

The turbidimetric method described by Pearce and Kinsella [51] was used to evaluate the emulsification activity index (ESI). Approximately 60 mg of sample was weighed and dispersed in 6 mL phosphate buffer (0.01 M, pH 7.0). After stirring 30 min at room temperature, soybean oil (2 mL) was added to the suspension. The protein in the buffer solution was homogenized for 1 min to form a homogenous emulsion and 50 μL from the bottom was added into a test tube containing 5 mL of 0.1% SDS solution (*w/v*) and mixed immediately. The absorbance of the solutions was measured using a Model UV-1601 spectrophotometer (Shimadzu, Kyoto, Japan) at 500 nm (*A*_0_). After 10 min, another 50 μL of emulsion were mixed with 5 mL of 0.1% SDS solution, and the absorbance of the mixture measured at 500 nm (*A*_10_). Emulsification activity index (*EAI*) and emulsion stability (*ES*) were expressed by the following Equations:

(3) $$EAI(m^{2}/g)=\frac{2\times 2.303\times A_{0}\times DF}{C\times \phi \times (1-\theta )\times 10000}$$

where *C* is protein concentration before diluted (g/mL), ϕ is the optical distance (1 cm), and *θ* is the oil volume fraction in the emulsion (0.25), *DF* is dilution ration (100).

The emulsion stability (*ES*) was determined by measuring the absorbance of emulsion after 10 min. *ES* as calculated by the following Equation:

(4) $$ES(\min)=\frac{A_{0}}{A_{0}-A_{10}}\times 10$$

#### 3.3.5. Surface Hydrophobicity (H_0_)

The H_0_ was determined with the fluorescence probe ANS- according to the method used by And and Lichan [52]. The samples were dissolved in 0.01 M phosphate buffer (pH 7.0) to obtain a protein concentration of 1 mg/mL. The stock protein solution was diluted to a final concentration of 0.002–0.01% (*w/v*) using the same buffer. ANS-solution (8.0 mM) was also prepared in the 0.01 M phosphate buffer (pH 7.0). For determination, 20 μL of ANS-solution was added to 4 mL of each dilution, and the fluorescence intensity (FI) of the mixture was measured at 390 nm (excitation) and 470 nm (emission) using a F-4500 fluorescence spectrophotometer (Hitachi Company, Tokyo, Japan). The initial slope of the FI versus protein concentration (mg/mL) plot (calculated by linear regression analysis) was used as an index of H_0_.

#### 3.3.6. Amino Acid Composition

The amino acid composition of protein samples were determined according to the method previously described by Du et al. [20]. Protein samples were hydrolyzed with 6 M HCl under a nitrogen atmosphere for 24 h at 110 °C, 20 μL of hydrolysate were loaded into an L-8800 automatic amino acid analyser (Hitachi). Amino acid compositions were expressed as g/100 g protein.

#### 3.3.7. Sodium Dodecyl Sulfate-Polyacrylamide Gel Electrophoresis (SDS-PAGE)

SDS-PAGE was carried out by the method of Malomo and Aluko [22]. Samples were treated by mixing with loading buffer containing 0.5 M Tris-HCl, glycerol, 0.1% bromophenol blue, 10% SDS only (non-reducing buffer) or 10% SDS and β-mercaptoethanol (reducing buffer) at ratio of 1:1 (*v/v*), then boiled at 100 °C for 3–5 min. After cooling and centrifugation, 10 μL of the treated samples were loaded on the 5% stacking gel and 12% separating gel, with electric current of 8 mA and 16 mA. When electrophoresis was over, the gels were stained with 0.025% Coomassie brilliant blue R-250 in 25.2% methanol and 6.4% glacial acetic acid for 30 min, then destained with 5% methanol and 7.5% glacial acetic acid until the background was near colourless. The molecular weight markers used were 14.4, 18.4, 25.0, 45.0, 66.2 and 116.0 kDa.

#### 3.3.8. Far-UV Circular Dichroism (CD) Spectroscopy

Far-UV CD spectroscopy was carried out to calculate the secondary structure of samples using a MOS-450 spectropolarimeter (French Bio-Logic SAS Co., Claix, France). Protein was dissolved in the 0.01 M phosphate buffer (pH 7.0) and the concentration was confirmed by Lowry’s method to obtained 0.1 mg/mL protein concentration. The CD spectroscopy was measured using quartz cuvette with path length of 1 mm, 100 nm/min scan rate, 1.0 nm of bandwidth, and 8 scans scanning from 190–250 nm. A mean residue weight of 110 was assumed in calculating the secondary structure and the data were expressed as mean residue ellipticity. The secondary structure compositions of the samples were estimated from the far-UV CD spectra using the CONTIN programe in Dichro Web.

#### 3.3.9. Scanning Electron Microscopy

The microstructure analysis was performed according to the method of Zhang et al. [53] using environmental scanning electron microscopy (SEM) (Quanta 200 F, FEI, Deutschland GmbH, Kassel, Germany). Briefly, protein samples were taken after lyophilization and sprayed with gold, then the samples were viewed at 5 kV and 3.0 spot sizes in the low vacuum mode to obtain the micro-morphology.

## 4. Statistical Analysis

All experiments were determined triplicate (CD determined eight times) and data were expressed as mean values ± standard deviation (SD). The date was subjected to correlation analysis using SPSS 18.0 (SPSS Inc., Chicago, IL, USA). *p* values < 0.05 were considered significant.

## 5. Conclusions

The molecular and functional properties of protein fractions and isolate from cashew nut kernel were investigated. The properties of the albumin, globulin, glutelin and CNPI fractions were considerably different. Globulin had a higher yield and purity than the other proteins. Cashew nut proteins were nutritionally equipped with abundant essential amino acids. SDS-PAGE indicated cashew nut proteins consisted mainly of polypeptides with ≤53 kDa, albumin and globulin showed more small polypeptides. The main secondary structure of cashew nut protein was β-sheets. Different solvent extraction procedures could change the structure of the proteins and led to the changes of their functional properties. Cashew proteins were observed to have the lowest solubility between pH 3.0 and 5.0, albumin and globulin almost fully dissolved in a neutral environment. Although CNPI showed the lowest solubility, it was found to have remarkable emulsifying properties and the highest H_0_ value. Glutelin had the best water/oil absorption capacity and foaming properties due to its amphipathic nature. This work investigated the molecular and functional properties of cashew nut proteins, and revealed that protein structure was an important factor for the functional properties. Different functional properties of cashew nut protein could be achieved by different production methods. NaOH-soluble glutelin exhibits superior water/oil holding capacity makes it suitable for cake, sausage and meat products. Water-soluble albumin with excellent solubility and emulsifying properties is strongly recommended for drinks. The results suggested that cashew nut proteins were potentially excellent protein sources for the food industry, meanwhile providing useful information for the development and utilization of global cashew nuts.

## Figures and Tables

**Table 1:** Proximate composition of defatted cashew nut flour, protein fractions and isolate.

Chemical Component (%)	Albumin	Globulin	Glutelin	Prolamin	CNPI	DCNF
Protein content	83.6 ± 0.8 ^b^	95.5 ± 1.1 ^a^	81.3 ± 3.8 ^b^	20.5 ± 2.3 ^d^	80.7 ± 1.3 ^b^	31.9 ± 2.1 ^c^
Crude fat	2.80 ± 0.39 ^a^	0.84 ± 0.38 ^b^	0.23 ± 0.04 ^b^	--	0.36 ± 0.13 ^b^	0.50 ± 0.36 ^b^
Moisture	4.55 ± 0.01 ^c^	2.18 ± 0.10 ^d^	4.49 ± 0.46 ^c^	--	8.87 ± 0.04 ^b^	9.24 ± 0.01 ^a^
Soluble sugar	2.03 ± 0.09 ^bc^	0.83 ± 0.13 ^c^	3.05 ± 0.32 ^b^	--	3.25 ± 0.24 ^b^	19.15 ± 1.21 ^a^
Ash	5.70 ± 0.20 ^a^	3.91 ± 0.80 ^b^	4.06 ± 0.69 ^ab^	--	4.61 ± 0.72 ^ab^	4.46 ± 0.16 ^ab^
Gross yield	7.69	17.30	7.80	2.5	19.67	--

All data were presented as mean value ± standard errors, *n* = 3. Different letters (^a–d^) in the same parameters indicated significant difference (*p* < 0.05), which was analyzed by SPSS software.

**Table 2:** Amino acid composition of cashew nut albumin, globulin and glutelin compare with CNPI from cashew nut (g/100 g protein).

Amino Acid	Albumin	Globulin	Glutelin	CNPI	FAO/WHO	
					Child	Adult
Aspartic acid (Asp)	6.52	8.31	7.65	8.10		
Threonine (Thr) ^a^	2.37	2.97	3.83	2.81	3.40	0.90
Serine (Ser)	4.15	5.04	4.37	4.16		
Glutamic acid (Glu)	22.51	22.55	12.57	21.54		
Glycine (Gly)	3.55	4.15	4.10	3.63		
Alanine (Ala)	2.37	2.97	4.37	2.90		
Cysteine (Cys)	2.37	1.78	0.82	0.75		
Valine (Val) ^a^	4.15	5.04	4.92	4.27	3.50	1.30
Methionine (Met) ^a^	1.78	1.19	1.64	1.15		
Isoleucine (Ile) ^a^	2.96	3.56	3.28	3.17	2.80	1.30
Leucine (Leu) ^a^	5.33	6.23	6.56	5.55	6.60	1.90
Tyrosine (Tyr)	2.96	2.97	3.28	2.26		
Phenylalanine (Phe) ^a^	3.55	3.56	3.83	3.42		
Histidine (His) ^a^	2.96	3.26	3.28	1.67	5.80	1.60
Lysine (Lys) ^a^	3.55	3.56	5.47	3.14	1.90	1.60
Arginine (Arg)	10.66	12.17	6.01	9.07		
Proline (Pro)	2.66	2.37	3.83	3.96		
sulphur amino acids	4.15	2.97	2.46	1.90	2.50	1.70
aromatic amino acid	6.51	6.53	7.11	5.68	5.68	1.90
hydrophobic amino acid	26.35	29.07	32.53	28.05		
EAA/TAA (%)	31.58	32.04	41.11	30.88		

^a^ Essential amino acids; Sulphur amino acids: methionine, cysteine; Aromatic amino acids: phenylalanine, tyrosine; Hydrophobic amino acids: alanine, valine, isoleucine, leucine, phenylalanine, proline, glycine; EAA/TAA: ratio of essential to total amino acids.

**Table 3:** Secondary structure composition of albumin, globulin, glutelin and CNPI from cashew nut.

Protein Sample	α-Helix (%)	β-Sheet (%)	Turns (%)	Random Coil (%)
Albumin	9.5 ± 0.15 ^b^	36.4 ± 0.24 ^b^	18.6 ± 0.16 ^b^	35.5 ± 0.24 ^a^
Globulin	6.8 ± 0.13 ^c^	41.1 ± 0.31 ^a^	22.5 ± 0.17 ^a^	29.5 ± 0.28 ^c^
Glutelin	11.7 ± 0.19 ^a^	36.1 ± 0.28 ^b^	18.3 ± 0.14 ^b^	33.9 ± 0.31 ^b^
CNPI	11.9 ± 0.43 ^a^	35.0 ± 0.24 ^b^	23.8 ± 0.17 ^a^	29.3 ± 0.11 ^c^

All data are represented as the mean value ± standard errors. Values in the same column with different letters (^a–c^) are significantly different (*p* < 0.05), as analyzed by the SPSS software.

**Table 4:** Water/Oil holding capacity of cashew nut protein fractions compare with CNPI in neutral environment.

W/O Holding Capacity	Albumin	Globulin	Glutelin	CNPI
WHC (g/g)	1.22 ± 0.02 ^b^	0.93 ± 0.13 ^b^	15.85 ± 1.67 ^a^	1.75 ± 0.05 ^b^
OHC (g/g)	5.94 ± 0.69 ^c^	9.34 ± 0.58 ^b^	27.47 ± 2.97 ^a^	1.05 ± 0.05 ^d^

All data are represented as the mean value ± standard errors. Values in the same line with different letters (^a–d^) are significantly different (*p* < 0.05), as analyzed by the SPSS software.

**Table 5:** Emulsifying and foaming properties of cashew nut protein fractions compare with CNPI in neutral environment.

Properties	Albumin	Globulin	Glutelin	CNPI
EAI (m^2^/g)	10.46 ± 0.27 ^b^	7.98 ± 0.75 ^c^	7.03 ± 0.33 ^c^	20.21 ± 1.53 ^a^
ES (min)	41.84 ± 1.97 ^b^	28.08 ± 1.16 ^c^	21.57 ± 2.20 ^d^	61.78 ± 4.44 ^a^
FC (%)	20.48 ± 2.02 ^c^	54.05 ± 3.67 ^b^	101.93 ± 16.39 ^a^	92.00 ± 1.15 ^a^
FS (%)	19.50 ± 0.93 ^c^	46.00 ± 9.63 ^b^	79.18 ± 5.82 ^a^	76.70 ± 5.77 ^a^

All data are represented as the mean value ± standard errors. Values in the same line with different letters (^a–d^) are significantly different (*p* < 0.05), as analyzed by the SPSS software.
